# Neuropsychological Performance and Cardiac Autonomic Function in Blue- and White-Collar Workers: A Psychometric and Heart Rate Variability Evaluation

**DOI:** 10.3390/ijerph20054203

**Published:** 2023-02-27

**Authors:** Ardalan Eslami, Najah T. Nassif, Sara Lal

**Affiliations:** 1 Neuroscience Research Unit, School of Life Sciences, University of Technology Sydney, Sydney 2007, Australia; 2 Neuroscience Research Unit, Transdisciplinary School, School of Public Health, University of Technology Sydney, Sydney 2007, Australia; 3 School of Life Sciences, University of Technology Sydney, Sydney 2007, Australia; 4 Honorary, School of Psychology, Faculty of Science, University of New South Wales, Sydney 2052, Australia

**Keywords:** heart rate variability, working memory, executive function, cognitive performance, autonomic nervous system

## Abstract

The 21st century has brought a growing and significant focus on performance and health within the workforce, with the aim of improving the health and performance of the blue- and white-collar workforce. The present research investigated heart rate variability (HRV) and psychological performance between blue and white-collar workers to determine if differences were evident. A total of 101 workers (*n* = 48 white-collar, *n* = 53 blue-collar, aged 19–61 years) underwent a three lead electrocardiogram to obtain HRV data during baseline (10 min) and active (working memory and attention) phases. The Cambridge Neuropsychological Test Automated Battery, specifically the spatial working memory, attention switching task, rapid visual processing and the spatial span, were used. Differences in neurocognitive performance measures indicated that white-collar workers were better able to detect sequences and make less errors than blue-collar workers. The heart rate variability differences showed that white-collar workers exhibit lower levels of cardiac vagal control during these neuropsychological tasks. These initial findings provide some novel insights into the relationship between occupation and psychophysiological processes and further highlight the interactions between cardiac autonomic variables and neurocognitive performance in blue and white-collar workers.

## 1. Introduction

In the 21st century, productivity is a crucial element in the strength and sustainability of a company’s gross business performance [1]. Both white-collar and blue-collar professions often require executive function to perform the tasks required for their work. However, compared to white-collar workers, blue-collar employees have been shown to have a higher prevalence of a large range of health complications, particularly cardiovascular disease (CVD) [2]. The workplace can often play a major role in the onset of cardiovascular disease and the current European guidelines on the prevention of CVD recommend an assessment of long-term stress, which includes occupational psychological stressors [3].

Executive cognitive function refers to a family of mental processes that are recruited for concentration and attention [4]. These executive functions have also been implicated in other aspects of health, such as obesity [5], occupational prosperity [6], and public safety [7]. Increasing evidence suggests an association between CVD and reduced psychological performance; however, few recent studies have delved into the inner workings which relate working memory (WM) to CVD. Additionally, many previous studies linking memory and working memory deficits to cardiac failures have mostly focused on patients with severe CVD [8].

Heart rate variability (HRV) has been extensively used to reflect the sympathetic and parasympathetic activity of the autonomic nervous system [9]. Furthermore, previous research has linked HRV to CVD [10,11], as well as various psychological processes [12,13]. Hansen et al. [12] established a relationship between HRV and performance tasks that taxed executive function in normal subjects (*n* = 53 male, average age = 23 years) and found that the qualitative differences between task demands could be predicted by the subject’s cardiac vagal tone. Other researchers have investigated this connection, but vagal tone relationships remain largely unexplored [14]. Furthermore, in order to predict cognitive performance by utilising cardiac vagal tone as an independent variable, Johnsen et al. [15] investigated attentional bias in 20 patients with anxiety in a dental setting using a modified Stroop-test [16] (14 male and 6 female, mean age = 36 years). Results showed that poor attentional performance was characterized by reduced HRV as compared to patients with higher HRV [15].

This indication of decreased HRV with increased working memory load and higher HRV in better performers supports the notion that, during working memory function, HRV may qualitatively predict cognitive differences among individuals [17]. This also implies that executive performance and autonomic functions, such as HRV, may be adaptively regulated by an interrelated neural network. Therefore, HRV may provide an index of an individual’s ability to function effectively in a dynamic environment [17].

Limited research has linked working memory and attentional deficits to cardiac deficits [18], with most studies focused on end stage patients [19]. Therefore, more research needs to be centred around healthy individuals, which may implicate HRV as a pre-emptive biomarker for working memory and attentional performance.

This study aims to investigate neuropsychological processes (working memory and attention) in two major working populations, white-collar (*n* = 48) and blue-collar (*n* = 53) workers, further identifying the fundamental associations between working memory, attention, and HRV. Heart rate variability and executive function are evaluated in a sample of healthy blue and white-collar workers to better understand the cardiac autonomic vagal influence during neuropsychological performance and risk factors that may contribute to cardiovascular complications. It was hypothesized that (1) attentional states will increase cardiac vagal input, HF and RMSSD HRV in white-collar workers while indicating a decrease in blue-collar workers, and (2) spatial neuropsychological stress will exhibit a decrease in cardiac vagal input, HF and RMSSD HRV in white-collar workers and an increase in blue-collar workers.

## 2. Materials and Methods

### 2.1. Participant Recruitment

Healthy participants between the ages of 18–68 years (*n* = 101) were recruited from the community. Participants were required to abstain from caffeine and nicotine for 4 h and alcohol for 12 h prior to the commencement of testing. These factors are known to influence physiological measures and their restrictions enhance the reliability of the data. Additionally, participants with pre-study blood pressure (BP) measures greater than 160 mmHg (systolic)/or 100 mmHg (diastolic) were excluded [20]. Testing was conducted between 8:30 am and 12:00 pm to minimize the effect of circadian rhythm fluctuation [21] on the data obtained. No volunteer was excluded from the current study and written informed consent was obtained prior to commencement of the study protocol. This study was approved by the Institutional Human Research Ethics Committee of the University of Technology Sydney (HREC: 2014000110 and HREC ETH19-3676).

### 2.2. Experimental Methodology

Participants were seated for 5 min prior to three BP recordings using an automated monitor (OMRON IA1B, Kyoto, Japan). Three blood pressure readings were obtained both before and after the study protocol with 2-min intervals between each measurement [22]. Participants were then asked to complete the General Health Questionnaire (GHQ60) [23], which obtained detailed health information. Participants then underwent a baseline electrocardiogram (ECG) for 10 min followed by an ECG recording during the neurocognitive tasks performed. The ECG was obtained using a FlexComp Infiniti encoder (Thought Technology Ltd., Montreal, QC, Canada) and an ECG-Flex/Pro amplifier sensor (Thought Technology Ltd., Canada) connected to three electrode leads. BioGraph Infiniti software (T7900) (Thought Technology Ltd., Canada) was used to record and display the ECG wave. Prior to placement of the electrodes, the skin was cleaned using Liv-Wipe (Livingstone International Pty Ltd., Sydney, Australia) 70% alcohol swabs. Disposable electrodes were used in all cases (Ag/AgCl ECG electrodes (Red Dot ^TM^) 2239, Tukwila, WA, USA).

The electrodes were placed in an inverted triangle to allow for positive deflections corresponding to the P, Q, R, S, and T waves [24]. The negative electrode was placed beneath the right clavicle, the ground electrode was placed beneath the left clavicle, and the positive electrode was placed 2 centimeters beneath the sternum and over the xyphoid process. Additionally, the ECG was sampled at 2048 samples per second for high precision detection of successive heart beats [25].

#### 2.2.1. Neuropsychological Tasks

The tasks performed utilized the Cambridge Neuropsychological Test Automated Battery (CANTAB) and tests included were the spatial working memory (SWM) task, attention switching task (AST), rapid visual processing task (RVP), and the spatial span (SSP) task [26]. The SWM task requires the retention and manipulation of visuospatial information. Outcome measures include errors, strategy, and latency. The AST is a test of a participant’s ability to shift attention between tasks and to ignore irrelevant information during interfering and distracting events. This test measures top-down cognitive control and provides measures of latency and errors. The RVP task is a measure of sustained attention assessing latency, probability, and sensitivity to pattern recognition. Finally, the SSP task is an assessment of working memory capacity and provides outcome measures of span length, errors, attempts, and latency.

#### 2.2.2. Heart Rate Variability

Prior to statistical analysis, ECG data was pre-processed to obtain time and frequency parameters of heart rate variability (HRV). The ECG data was imported into Kubios HRV software (Version 3.1, University of Kuopio, Kuopio, Finland). The R-waves were automatically detected by applying the built-in QRS detection algorithm [27]. Frequency bands obtained were low frequency (LF) (0.04–0.15 Hz), high frequency (HF) (0.15–0.4 Hz), total power HRV (TP), and the ratio of LF to HF (LF/HF). The inbuilt process within Kubios and the smoothness priors method was used to correct for artefacts and ectopic beats in the raw ECG data [27,28]. It should also be noted that the data were log-transformed prior to analysis, where relevant.

### 2.3. Statistical Analysis

Statistical analysis was performed using SPSS Version 22.0 (IBM Corp., 2013, New York, NY, USA) IBM Corp [29] with statistical significance reported at *p* < 0.05. Independent sample *t*-tests were applied to establish significant differences in HRV parameters and neurocognitive performance measures between the blue and white-collar workers.

## 3. Results

### 3.1. Demographic Data of Blue and White-Collar Workers

The demographic data of the blue and white-collar groups are shown below in Table 1. Compared to the blue-collar workers, the white-collar workers had spent significantly more time in education (3.4 ± 1.2 years and 4.33 ± 1.2 years, respectively) (*p* < 0.001).

### 3.2. Neuropsychological Performance of Blue and White-Collar Workers

Independent sample *t*-tests of neuropsychological performance showed significant differences in the tasks (SWM, AST, RVP, SSP) between white (*n* = 48) and blue-collar (*n* = 53) workers. The significant findings are presenting in Table 2.

Attention Switching Task: During the AST, the white-collar sample group were found to make fewer errors when incongruent cues were given compared to the blue-collar worker group (8 ± 3.14 and 9.4 ± 3.62, respectively) (*p* = 0.04). When the “side” cue was given, the white-collar worker group made more errors than the blue-collar worker group (4.33 ± 2.56 and, 3.19 ± 2.16, respectively) (*p* = 0.02). Moreover, the white-collar worker group made significantly more correct responses than the blue-collar worker group overall (144.20 ± 8.53 and, 139.43 ± 9.80, respectively) (*t* = *p* = 0.01).

Rapid Visual Processing Task: Throughout the RVP task, the ability to detect signals was significantly higher in the white-collar worker group as compared to the blue-collar worker group (0.90 ± 0.08 and, 0.85 ± 0.08, respectively) (*p* = 0.002).

Spatial Span Task: Finally, the SSP task saw the white-collar worker group make more total errors than the blue-collar worker group (13.48 ± 5.65 and, 9.74 ± 6.55, respectively) (*p* = 0.003).

### 3.3. HRV in Blue and White-Collar Workers

Independent sample *t*-tests were used to compare HRV parameters between the white and blue-collar worker groups. The significant findings are summarised in Table 3.

Spatial Working Memory Task: Throughout the SWM task, the white-collar worker group, compared to the blue-collar worker group, had significantly higher log LF (6.33 ± 0.60 and 6.01 ± 0.48, respectively) (*p* = 0.004), log LF/HF (1.61 ± 0.83 and 1.22 ± 0.84, respectively) (*p* = 0.02), and log TP (6.7 ± 0.52 and 6.48 ± 0.37, respectively) (*p* = 0.02).

Rapid Visual Processing Task: The RVP task highlighted significantly lower HRV parameters in the white-collar worker group compared to the blue-collar worker group, particularly log LF (6.16 ± 0.47 and 6.44 ± 0.33, respectively) (*p* < 0.001), log HF (5.04 ± 0.60 and 5.28 ± 0.42, respectively) (*p* = 0.03), log TP (6.61 ± 0.44 and 6.89 ± 0.27, respectively) (*p* < 0.001), and log SDNN (3.39 ± 0.22 and 3.53 ± 0.12, respectively) (*p* < 0.001).

Spatial Span Task: During the Spatial Span (SSP) task, it was found that log HF was significantly lower in the white-collar worker group as compared to the blue-collar worker group (4.81 ± 0.58 and, 5.07 ± 0.67, respectively) (*p* = 0.03).

## 4. Discussion

The present study aimed to investigate the differences in HRV and psychological performance between a sample of blue and white-collar workers. The analysis indicated higher vagal cardiac mediation in blue-collar workers, as indexed by RMSSD and HF HRV, in response to spatial working memory and attention based cognitive tasks. Additionally, these results show that blue-collar workers performed significantly better on spatial tasks while white-collar workers performed better on attentional process tasks.

The current literature comparing these two sub groups is very limited; however, early work by Myrtek [29] investigating the level of stress and strain and its relationship to heart rate, physical activity, emotional strain, and mental strain found no differences in variability of heart rate (HR) between the two groups. The authors did, however, find that white-collar workers were more stressed, subjectively [29]. Additionally, it is thought that blue-collar workers are subject to an increased physical workload while white-collar workers are thought to have a high mental workload, and although interviews and questionnaires supported this idea, the physiological measurements did not [29].

Early work in the literature highlights conflicting evidence regarding the predisposition of blue and white-collar workers to CVD with some studies suggesting blue-collar workers were more at risk [30] while others suggested white-collar workers were more at risk [2]. Moreover, there is very little research investigating HRV parameters, psychological performance measures, and their associations with CVD in these two cohorts, and the present research aimed to provide more information and data regarding the relationship between different occupational and physiological risk measures and CVD.

When comparing the two sample cohorts, the only statistically significant difference in demographics was the years spent in education, where the blue-collar workers had spent less time in education than the white collar workers. Interestingly, Prihartono et al. [2] found that the increased level of education of white-collar workers significantly increased the prevalence of CVD. Moreover, prevalence of CVD by diagnosis was higher in the white-collar worker population, while the prevalence by symptoms was higher among the blue-collar worker group [2]. Even though the blue-collar workers are inherently more physically active in their day to day work, their socio-economic status and lifestyle choices may have a significant impact, particularly in the available access to health care. Lower education and lower salaries are more likely to predispose to unhealthy lifestyle choices [31]. Moreover, a higher BMI increased the prevalence of CVD in both blue and white-collar workers [2].

### 4.1. Heart Rate Variability Parameters during Neuropsychological Performance

#### 4.1.1. Spatial Working Memory

During the SWM task, the LF, LF/HF, and TP parameters of HRV were all greater in the white-collar worker group compared to the blue-collar worker group. LF HRV was traditionally thought to reflect sympathetic activity, as previously mentioned, but recent research indicates it is influenced by both the sympathetic and parasympathetic branches of the ANS [32]. This increase in LF HRV activity may point to increased sympathetic activity and dominance during these tasks for the white-collar worker group. This finding has been previously associated with an increased risk of CVD [33]. This has also been contrasted by other literature reporting that low LF HRV was associated with certain risk factors which predispose to CVD, for example, hypertension [34]. Moreover, a review by Hillebrand et al. [35] highlighted that low HRV indices, including LF HRV, indicated a higher risk of CVD in populations without any prior CVD. Interestingly, much of the prior research indicates that vagal withdrawal, and therefore an increased sympathetic response, is responsible for the cardiovascular disease risks [36]. However, Hamaad et al. [33] provide a differing perspective, suggesting that it is sympathetic activation which may be associated to cardiac events, and not the former. The authors of [33] investigated the associations between indices of HRV (time and frequency) and inflammatory biomarkers in patients with acute coronary syndrome (*n* = 100, male = 77, average age = 63 ± 12 years) and healthy controls (*n* = 49, male = 32, average age = 60 ± 10 years). Though the correlations were modest, the authors reported an inverse relationship between LF HRV and inflammatory biomarkers and, therefore, implicate sympathetic tone in CVD [33]. This idea is further supported by several studies which further investigate the inflammatory biomarkers and associated HRV changes [37].

#### 4.1.2. Rapid Visual Processing

The RVP task showed that the blue-collar workers had higher HRV parameters across the board, particularly LF, HF, TP and SDNN. This is an interesting finding as the RVP task is one of sustained attention, and it was therefore expected that the white-collar worker group would exhibit higher levels of cardiac vagal control, as indexed by HF HRV or RMSSD. The findings of the present research may reflect high levels of stress within the white-collar working population as shown by lower HF HRV. Previous research concluding which occupational group is more stressed is contentious, and the literature suggests a multitude of variables that may contribute. Dedele et al. [38] indicate that blue-collar workers are 1.5 times more likely to perceive higher levels of stress in general. However, the white-collar workers had a four times increased likelihood of perceiving greater stress when they had been sedentary for more than 3 h per day [38]. Contrastingly, Nydegger [39] found no significant differences in stress levels between blue and white-collar workers, nor any differences between genders. Given that these studies only assessed perceived stress by way of surveys, the results may be too subjective, with numerous factors potentially influencing the responses. The use of a more objective measure would have been of great benefit to support their findings. Notwithstanding, they do provide grounds to indicate intricate interrelationships between workplace stress, HRV, and CVD. Moreover, recommendations made to white-collar workers include making improvements in sedentary lifestyle and increasing physical activity during work hours, while blue-collar workers must avoid unhealthy lifestyle habits [39,40]. These practices will ultimately reduce stress, improve cardiac autonomic activity and parasympathetic input, and therefore may reduce the risk of a cardiovascular event.

#### 4.1.3. Spatial Span

The final difference in HRV between the blue and white-collar worker group found in this study was related to the SSP task, whereby the blue-collar worker group showed higher vagal mediation than the white-collar worker group. This is indicative of better control and better performance. Moreover, it may indicate a more relaxed scenario, as the SSP task is designed to evaluate working memory capacity in the 3D space around them, an environment familiar to blue-collar workers.

### 4.2. Comparison of Neuropsychological Performance between Blue and White-Collar Workers

Occupation has been considered as an important predictor of cognitive ability and decline over time [41]. Furthermore, the executive function requirements in the workplace, as well as the complexities of the environment, seem to have a correlation to cognitive decline [42]. Prior research has tended to be focused on age-related decline in cognitive processing and few studies have focused on the occupational effects. However, given that people spend a substantial portion of life at work, the workplace environment may have a significant effect [43].

#### 4.2.1. Attention Switching

The AST showed that the white-collar workers made less errors when the cues were changing and more errors when the “side” cue was given. However, in the task as a whole, the white-collar workers gave significantly more correct responses than the blue-collar workers. In a longitudinal study spanning 10 years, Kim et al. [44] assessed executive function in blue (*n* = 1216, 61% Female, aged 70.7 ± 4.64 years) and white-collar workers (*n* = 242, 22% Female, aged 69.98 ± 4.18 years). The authors gathered data using the Mini-Mental Sate Examination (MMSE) [45] and other potential covariates, including sociodemographic factors, health related factors and occupational factors [44]. Primary findings between the longest-held lifetime occupation and executive function decline showed that males had no significant risks, whilst females showed a 2.5-fold increased risk of cognitive impairment amongst blue-collar workers compared to white-collar workers [44].

#### 4.2.2. Rapid Visual Processing/Spatial Span

The white-collar workers showed significantly better performance during the RVP task, where their ability to detect sequences was much better. However, the white-collar workers made more errors during the SSP task. The relationship between mental workload and cardiovascular parameters is further illustrated by Capuana et al. [46]. These authors assessed 22 young adults (17 women, 18–27 years, average age = 20.5 years (SD not specified)) and 18 older adults (11 women, 65–83 years, average age = 72.3 (SD not specified)) and indicated relationships between cardiac measures and performance, as well as an association between increased cardiac workload and more errors in the older adults but not the younger adults [46]. This further supports and adds to the age-related literature regarding neurocognitive performance with the added element of cardiac risk measures. The results of previous literature and the present findings suggest that the effects of occupation on executive functions are multifaceted [41]. Prior research has indicated that white-collar workers are more cognitively inclined in the later years [41]. Moreover, manual labor workers (including machine operators, assembly workers and plant operators) have been shown to have a significantly higher chance of reduced executive function as compared to non-manual laborers (including business executives, administrators, and managers) [47]. As a whole, the white-collar workers seem to have performed better on the executive function tasks. Notwithstanding the varying performance on different tasks, an in depth analysis must be conducted to supplement broader examinations in order to identify specific relationships between cardiac variables and neurocognitive performance measures.

Several factors may be considered when assessing the performance and risks between the blue and white-collar worker populations. Most people spend a large portion of their life at work, and so the inherent risks related to employment are something that must be further researched. These risks may be a result of the complexity in given occupations, which was first touched upon by Schooler [48] and further by Schooler et al. [49]. These authors suggested that complex environments at work, or during leisure time, allow for continued reinforcement of executive function. This greater intellectual stimulation increases neural growth and synaptic density, which protects against cognitive decline [50]. Therefore, lower intellectual demands for blue-collar workers may predispose them to executive function impairments. This is just one facet by which the literature suggests the enhanced ability of white-collar workers. Another theory indicates that, since blue-collar work is associated with a lower income, this translates to poor housing, nutrition, environment, and poor lifestyle habits and practices, which may be linked to cognitive decline [51,52]. Interestingly, white-collar workers are more educated in the traditional sense, but this does not necessarily reflect in overall intelligence. Given that white-collar workers are known to use cognitive abilities more often than blue-collar workers, it could be assumed that they have superior cognitive abilities. This may not be the case however, as a study showed that there was no evidence that regular use of computerized brain trainers improves general cognitive functioning [53].

### 4.3. Limitations and Future Directions

The present findings show perhaps that changes in HRV are in fact influenced by various tasks, spanning all professions. Increased sample numbers in each profession would allow for stratification and observations within the same job type. For example, one white-collar worker may perform more administrative tasks while another may perform more data analytics and these differences in neuropsychological load may further influence HRV. Moreover, this cross-sectional design provides a snapshot in time of the measures. Therefore, a longitudinal study would allow for a more in-depth analysis of how a particular profession may influence these physiological variables over the course of one’s life. It is also acknowledged that, even though only 18% of the blue-collar worker group was made up of female workers, this is an accurate reflection of this population sample [54]. Though the present study identified numerous findings, it may only be predictive in nature and not causal. Therefore, future studies may be able to investigate the causal link between vagal tone, working memory, and attention through various techniques, such as transcutaneous vagal nerve stimulation or other neuroimaging techniques.

## 5. Conclusions

Overall, the present research identified multiple significant differences in HRV parameters and neurocognitive performance measures between the blue and the white-collar workforce. Blue-collar workers indicated higher vagally mediated cardiac control during neuropsychological tasks with better performance in spatial working memory exercises, whilst white-collar workers had superior performance on attention-based tasks.

Notably, reduced parasympathetic modulation of the heart, particularly in white-collar workers, was observed.

## Figures and Tables

**Table 1:** Compilation of the demographic variables of the blue and white-collar sample groups.

Demographic	White-Collar (Mean ± SD)	Blue-Collar (Mean ± SD)	*p*
Years of Age	39.83 ± 10.98	36.85 ± 11.28	0.19
Male (*n*; %) Female (*n*; %)	*n* = 25 (52%) *n* = 23 (48%)	*n* = 42 (81%) *n* = 11 (19%)	
BMI	23.54 ± 2.40	22.7 ± 2	0.06
Education (years)	4.33 ± 1.20	3.4 ± 1.20	<0.001
GHQ Score	6.88 ± 2.08	6.9 ± 2	0.87

Key: BMI = Body mass index; GHQ = General health questionnaire 60 (Goldberg, 1972); *n* = Sample size; SD = Standard deviation; % = Percentage.

**Table 2:** Neuropsychological Performance Measures between the Blue (*n* = 53) and White-collar (*n* = 48) worker sample groups.

Task	Variable	F	df	*p*	White-Collar Workers Mean ± SD	Blue-Collar Workers Mean ± SD	Mean Difference (White-Blue)
AST	Incongruent Errors	1.25	99	0.04	8 ± 3.14	9.4 ± 3.62	−1.40
	Errors (side)	3.21	99	0.02	4.33 ± 2.56	3.19 ± 2.16	1.14
	Total Correct	0.03	99	0.01	144.20 ± 8.53	139.43 ± 9.80	4.77
RVP	Signal Detection	0.03	99	0.002	0.90 ± 0.08	0.85 ± 0.08	0.05
SSP	Total Errors	1.30	99	0.003	13.48 ± 5.65	9.74 ± 6.55	3.74

Key: AST = Attention switching task; df = Degrees of freedom; F = F statistic; *n* = Sample Size; *p* = Level of statistical significance (*p* < 0.05); RVP = Rapid Visual processing; SD = Standard deviation; SSP = Spatial Span.

**Table 3:** Differences in HRV Parameters between the White (*n* = 48) and Blue-collar (*n* = 53) worker sample groups.

Task	Variable	F	df	*p*	White-Collar Mean ± SD	Blue-Collar Mean ± SD	Mean Difference (White-Blue)
SWM	Log LF (ms^2^)	0.69	99	0.004	6.33 ± 0.60	6.01 ± 0.48	0.31
	Log LF/HF	0.66	99	0.02	1.61 ± 0.83	1.22 ± 0.84	0.39
	Log TP (ms^2^)	1.59	99	0.02	6.7 ± 0.52	6.48 ± 0.37	0.22
RVP	Log LF (ms^2^)	5.41	79.87	<0.001	6.16 ± 0.47	6.44 ± 0.33	−0.29
	Log HF (ms^2^)	7.84	78.50	0.03	5.04 ± 0.60	5.28 ± 0.42	−0.233
	Log TP (ms^2^)	11.72	74.72	<0.001	6.61 ± 0.44	6.89 ± 0.27	−0.28
	Log SDNN (ms)	11.1	−3.82	<0.001	3.39 ± 0.22	3.53 ± 0.12	−0.14
SSP	Log HF (ms^2^)	0.67	98	0.03	4.81 ± 0.58	5.07 ± 0.67	−0.27

Key: df = Degrees of freedom; F = F statistic; HF = High frequency; HRV = Heart rate variability; LF = Low frequency; LF/HF = Low frequency divided by high frequency; ms = Milliseconds; ms^2^ = Milliseconds squared; *n* = Sample Size; *p* = Level of statistical significance (*p* < 0.05); RVP = Rapid visual processing; SD = Standard deviation; SDNN = Standard deviation of all NN intervals (square root of variance); SSP = Spatial span; SWM = Spatial working memory; TP = Total power.

## Data Availability

All data relevant to the present study are available from the corresponding author on reasonable request.
